# Evaluation of black soldier fly larvae oil (*Hermetia illucens* L.) calcium salt as an alternative fat source for laying quail diets

**DOI:** 10.5713/ab.24.0289

**Published:** 2024-10-25

**Authors:** Hafidz Hukma Shabiyya Armaghoza, Setyo Nugroho, Sungkono Sungkono, Septiyanto Lukman Widodo, Bramasta Cahyo Saputra, Muhammad Anang Aprianto, Muhsin Al Anas

**Affiliations:** 1 Animal Nutrition and Feed Science Department, Faculty of Animal Science, Universitas Gadjah Mada, Yogyakarta, Indonesia; 2 Department of Anatomy, Faculty of Veterinary Medicine, Universitas Gadjah Mada, Yogyakarta, Indonesia

**Keywords:** Black Soldier Fly Larvae Oil, Calcium Salt, Egg Quality, Gene Expression, Performance, Quail

## Abstract

**Objective:**

This study aims to determine the effect of adding saponified black soldier fly larvae oil calcium salt (BSFLO-SCa) to quail feed as an alternative source of fat on laying performance, blood lipid profile, egg quality, and gene expression in lipid metabolism.

**Methods:**

A total of 120 female Japanese quails (*Coturnix japonica*) aged 24 weeks were divided into 3 treatments, each with 8 replications, and each replication consisted of 5 quails in a completely randomized design. The applied treatments were the inclusion of basal feed as a control (T0) and basal feed supplemented with 1% BSFLO-SCa (T1) and 2% BSFLO-SCa (T2).

**Results:**

The study indicated that the supplementation starting from 1% of BSFLO-SCa significantly decrease (p<0.05) in feed conversion ratio, blood lipid profile (total cholesterol, low density lipoprotein-cholesterol). Gene expression on fat synthesis of fatty acid synthase and cholesterol synthesis of 3-hydroxy-3-methylglutaryl coenzyme A reductase downregulated (p<0.05). In addition, the other parameters did not affect by supplementation of 1% BSFLO-SCa. The inclusion at 2% of BSFLO-SCa significantly increased (p<0.05) protein content of yolk and albumen, egg weight, egg shape index, and gene expression on fat oxidation of carnitine palmitoyltransferase-1. Egg yolk cholesterol, egg albumen ash, haugh unit, and gene expression on fat synthesis of acetyl-CoA carboxylase were significantly reduced (p<0.05).

**Conclusion:**

Addition of 2% BSFLO-SCa in the feed improves performance, egg quality, and reduces cholesterol in the blood and eggs of quail. This improvement is accompanied by a reduction in the expression of key genes involved in lipid metabolism. BSFLO-SCa oil has the potential to be an alternative oil source in quail feed.

## INTRODUCTION

In Indonesia, quail eggs are popular among consumers as an alternative source of protein to chicken eggs due to their high nutritional value, relative affordability. However, quail eggs are known for their high cholesterol content. The cholesterol content in quail egg yolk is approximately 16.05 mg/g, which is higher compared to chicken eggs and duck eggs, which have cholesterol levels of around 7.65 mg/g and 10.36 mg/g, respectively [1]. Fatty acids that typically come from the addition of oil in the feed, will influence cholesterol formation through their metabolism into acetyl-CoA, which acts as a precursor for cholesterol [2].

The inclusion of oil in animal feed offers several benefits, such as minimizing dust formation, reducing particle segregation in mash feeds, enhancing palatability, serving as carriers for fat-soluble vitamins, providing essential fatty acids and aiding in the lubrication of feed milling equipment. Generally, crude palm oil (CPO) is used in feed due to its relatively low cost [3]. However, its high saturated fatty acid (SFA) content, with palmitic acid comprising 43%, has the potential to increase blood total cholesterol, high density lipoprotein (HDL) cholesterol levels, and low density lipoprotein (LDL) cholesterol level which also affects both meat and eggs [4]. Furthermore, the palm oil industry is linked to practices that are not environmentally sustainable, resulting in issues such as air pollution, soil degradation, and deforestation, which consequently lead to a reduction in biodiversity [5]. Among alternative oil sources, black soldier fly larvae oil (BSFLO) has emerged as a viable option due to its beneficial fatty acid profile (dominated by lauric acid) [6]. Although the current price of BSFLO is higher than CPO due to limited cultivation, prices are expected to adjust as the industry develops and the benefits become apparent. Additionally, the use of BSFLO offers a sustainable oil source and aids in organic waste management [6].

BSFLO is a product resulting from the extraction process of BSFL. The fatty acid composition of BSFLO is dominated by SFA, which include medium chain fatty acids (MCFA) dominated by lauric acid (C 12:0) accounting for up to 52% [6]. Lauric acid, which is classified as an SFA can indeed increase cholesterol levels. This increase occurs in HDL-cholesterol (good cholesterol), which actually transports excess cholesterol from the peripheral tissues to the liver through an increase in ApoA1 secretion, a main protein component of HDL-cholesterol [7]. MCFA effectively generate energy as they can directly enter the mitochondria without the carnitine transport mechanism and are primarily used for β-oxidation, which prevents lipid deposition in tissues [8]. Saeidi et al [9] reported that the addition of MCFA to quail feed reduced total cholesterol, LDL-cholesterol, triglycerides, and abdominal fat, while increasing HDL-cholesterol.

Recent studies reported that the use of BSFLO improved laying hens’ productivity, intestinal morphology, and integrity by enhancing tight junction protein expression and boosting intestinal immune responses through the modulation of inflammatory cytokine gene expression [10]. Other research reports that BSFLO supplementation in broiler chickens increases HDL-cholesterol and blood protein levels, decreases the expression of fat synthesis genes (acetyl-CoA carboxylase [ACC], fatty acid synthase [FAS]), and increases genes related to β-oxidation (carnitine palmitoyltransferase [CPT-1]) [11]. Several studies mentioned earlier form the basis of this research. However, information on the application of BSFLO in quail feed remains limited, particularly its effects on mRNA expression related to fat metabolism in laying quail. On the other hand, calcium saponified oil is produced through saponification. Forming granules that are easily mixed with feed and resistant to oxidation [12]. This study aimed to determine the effect of CPO replacement with black soldier fly larvae oil calcium salt (BSFLO-SCa) on performance, blood fat profile, egg quality, and gene expression associated with fat metabolism in laying quail.

## MATERIALS AND METHODS

### Animal care

The Faculty of Veterinary Medicine, Universitas Gadjah Mada’s research ethics committee reviewed and approved the experiment with ethical clearance No: 076/EC-FKH/Eks./2023.

### Animal and housing

A total of 120 Japanese quails (*Coturnix japonica*), aged 24 weeks, were weighed to determine their initial body weight (BW) and observed of egg production to ensure balanced distribution between treatment groups. Quails with equal BW (211±6.17 g) and similar levels of egg production were placed into treatment cages. The quails were randomly assigned, with a balanced treatment order, to 24 cages. Each cage, measuring 120×50×23 cm, housed 5 quails and was equipped with feeders and drinkers. A lighting program was provided for 16 hours period of light and 8 hours period of darkness. Indoor temperature was maintained at 27±2°C, while the relative humidity ranged from 60% to 65%. The treatment feed, containing 20% crude protein, was provided for two months (56 days), and water was provided ad libitum. When feeding, health monitoring of the quails is also carried out. Egg collection was conducted daily and the cages were cleaned weekly.

### Black soldier fly larvae oil and crude palm oil calcium salt processing

The BSFLO and CPO mixed in the feed were converted into solid form through saponification. The process of producing BSFLO-SCa involved reacting BSFLO from PT Magalarva Sayana in Banten, Indonesia, with 10.4% NaOH solution and stirring at a temperature of 70°C until it formed a solid. Subsequently, it underwent another reaction with 10.4% CaCl_2_ solution until a bright brown, crumbly solid was obtained according to the method described by Aprianto et al [11]. The same process was also applied crude palm oil calcium salt (CPO-SCa). The fatty acid compositions of BSFLO-SCa and CPO-SCa were analyzed using gas chromatography (GC; Agilent Technologies 7890B; Agilent Technologies, Santa Clara, CA, USA) (Table 1) following a modified method described by Mjøs [13]. The peaks in the chromatogram can be identified by their retention times and matched against commercial standards.

### Experimental design and diets

The experimental design employed in this study is a Completely Randomized Design with a one-way pattern consisting of 3 treatments with 8 replications, each replication containing 5 quails. The birds in each group were provided with a basic diet including containing saponified crude palm oil as a control (T0), and this was replaced with 1% (T1) and 2% (T2) of BSFLO-SCa. The treatment feeds were formulated with iso-caloric and iso-energic and to meet the nutritional requirements of laying quails based on the protocol by NRC [14] (Table 2). The fatty acid profiles of treatment diets were analyzed by GC (Agilent Technologies 7980B) following the procedure detailed by Mjøs [13], as presented in Table 3.

### Data collection

#### Performance and egg physic quality

During the 2-month treatment period, the amount of feed given and remaining feed were recorded weekly. Egg production was calculated, and egg weight (EW) was measured daily and calculated weekly. The data obtained were used for productivity analysis, including average daily feed intake (ADFI), feed conversion ratio (FCR), hen day average (HDA), egg mass (EM), and EW. EM was calculated by multiplying EW with HDA, while HDA was determined by dividing the overall egg production by the total number of quail, and then multiplying the result by 100. Physical quality testing of quail eggs was conducted on the last 3 days of each month’s treatment (at 26, 27, 28, 54, 55, and 56 days of age). Physical egg quality tests observed with parameter of EW, shape index, haugh unit (HU), yolk color, yolk height and width, albumen height and width, eggshell weight, and eggshell thickness [15]. The eggs used for physical quality testing were one egg from each replication of the treatment, ensuring that the EW is representative of the sample used. Yolk color measurement was performed using a roche fan, which has a 15-color scale.

#### Blood lipid profile

At the end of the experiment (56 days), blood serum samples were obtained from 18 birds. Each treatment group consisted of 6 birds, with one bird selected from each replicate. The chosen birds had BW close to the median for their respective groups. The birds were euthanized by decapitation, and jugular vein was cut to collect blood serum samples, which were then stored in Eppendorf tubes at a temperature of −20°C until analysis. The concentration of total cholesterol, HDL-cholesterol, LDL-cholesterol, and triglycerides was measured using UV-visible spectrophotometer (Microlab 200; Merck Vital Scientific, Darm-slat, Netherlands). Suitable with the commercial kits for veterinary use (DiaSys Diagnostic System GmbH, Holzheim, Germany).

#### Egg chemical quality

Quail eggs underwent separate chemical quality testing for both yolk and albumen at the end of the experiment (56 days). Egg samples were obtained from 18 birds, with 6 eggs from each treatments group and 1 egg per replication. The chemical quality analysis of both albumen and yolk included the content of dry matter, ash and crude protein, while crude fat was analyzed only in the yolk following the method outlined by Association of Official Analytical Chemistry International (AOAC) [16]. The cholesterol content in the yolk was determined using the Liberman-Burchard method as described by Shafiq et al [17].

#### Gene expression in quantitative real-time polymerase chain reaction lipid metabolism

Liver samples from 1 bird in each replicate (18 birds) were collected in a Eppendorf tubes, rapidly frozen in liquid nitrogen, and stored at −80°C until analysis. RNA extraction was initiated by processing liver samples weighing up to 30 mg using a Quick-RNA miniprep kit (Zymo Research Corp., Irvine, CA, USA), following established protocols. The RNA purity and quantity were assessed using a Nanodrop Spectrophotometer (Maestrogen Inc., Hsinchu, Taiwan). The extracted RNA was reverse transcribed using the Applied Biosystems ProFlex polymerase chain reaction (PCR) system (Thermo Fisher Scientific, Waltham, MA, USA) and ReverTrace quantitative PCR (qPCR) RT Master Mix (Toyobo Co., Ltd., Osaka, Japan) for cDNA synthesis.

The primary sequences used in this study are presented in Table 4 [18,19]. Gene expressions were performed in duplicates using the Quant-Studio 3 Real-Time PCR machine (Thermo Fisher Scientific) and Thunderbird SYBR qPCR Mix (Cat No. QPX-201; Toyobo Co., Ltd., Osaka, Japan), following established technique. In summary, a total of 1 microliter of diluted cDNA, 6 picomoles of forward primer, 6 picomoles of reverse primer, and 5 microliters of qPCR Mix were combined in a tube. Nuclease-free water was added to bring the total reaction volume to 10 microliters. The amplification procedure consisted of a hold stage at 95°C for 2 minutes, followed by a PCR stage with 40 cycles of 1 second at 95°C and 30 seconds at 60°C. At the conclusion of the run, the melt curve was examined to confirm amplification of the desired product. The mRNA levels were normalized by calculating the ratio to β-actin using the 2^−ΔΔC(t)^ method and the resulting data were then reported as relative values compared to the control group [20].

### Statistical analysis

All experimental data were analyzed statistically using IBM SPSS statistic version 26.0. The data was subjected to one-way ANOVA among three treatments. The results of the analysis aimed to determine the effects of BSFLO-SCa supplementation on performance, egg physic quality, blood lipid profiles, chemical quality of eggs, and the expression of fatty acid synthesis genes. Differences among treatments were examined using Duncan’s multiple range tests. The statistical significance of all analyses was set at p<0.05 for probability values.

## RESULTS

### Performance

The effect of BSFLO-SCa supplementation on productive laying performance is presented in Table 5. Supplementation with BSFLO-SCa significantly decreased FCR (p<0.05). Furthermore, the non-significant results (p>0.05) indicated that dietary supplementation of BSFLO-SCa did not have negative impact the productive performance parameters, such as HDA, EM, EW, and ADFI.

### Egg physical quality

Table 6 presents the effect of BSFLO-SCa supplementation on the physical quality of quail eggs. Dietary supplementation BSFLO-SCa at 1% did not statistically significant differences (p>0.05) compared to the control treatment in the egg physical quality. However, the BSFLO-SCa 2% diet significantly (p<0.05) increased EW, shape index, and decreased HU. Nevertheless, there were no significant differences (p>0.05) from the control observed in other parameters, including yolk color, yolk height and width, albumen height and width, eggshell weight, and eggshell thickness.

### Blood lipid profile

The effect of BSFLO-SCa supplementation on blood lipid profile is presented in Table 7. Triglyceride and HDL-cholesterol content did not differ significantly from the control (p>0.05). However, cholesterol and LDL-cholesterol content decreased significantly (p<0.05) with increased supplementation of BSFLO-SCa.

### Egg chemical quality

Table 8 shows the effect of BSFLO-SCa supplementation on the chemical quality of quail eggs. Dietary supplementation of BSFLO-SCa at 1% did not statistically significant (p>0.05) compared to the control treatment regarding the chemical quality of egg yolk and albumen. However, the 2% treatment significantly increased (p<0.05) the protein content in both the egg yolk and albumen. Additionally, the 2% treatment also significantly (p<0.05) reduced yolk cholesterol and albumen ash content. Nevertheless, supplementation with 2% did not significantly (p>0.05) affect yolk dry matter, ash, crude fat, and albumen dry matter content.

### Gene expression in lipid metabolism

**Figure 1:**
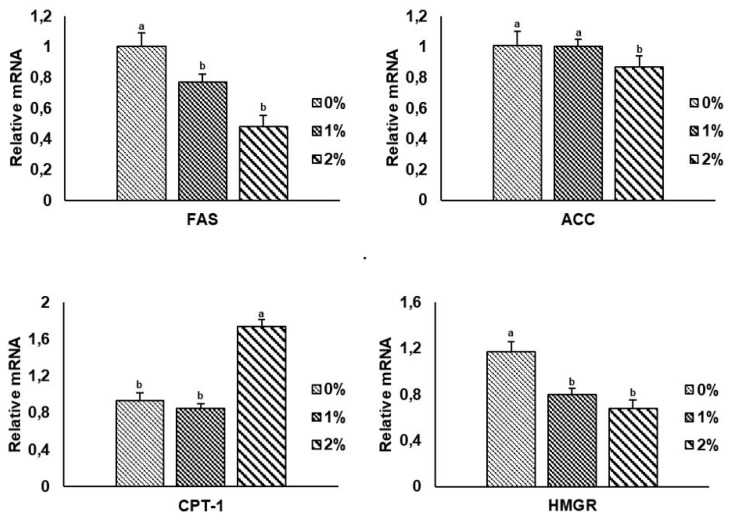
Comparison between FAS, ACC, CPT-1, and HMGR mRNA expression in Japanese quail livers fed with the supplementation of black soldier fly larvae oil calcium salt (BSFLO-SCa). ^a,b^ p<0.05. FAS, fatty acid synthase; ACC, acetyl-CoA carboxylase; CPT, carnitine palmitoyltransferase; HMGR, 3-hydroxy-3-methylglutaryl coenzyme A reductase; BSFLO-SCa, black soldier fly larvae oil calcium salt.

The effect of BSFLO-SCa supplementation on gene expression related to liver lipid metabolism is presented in Figure 1. The 2% BSFLO-SCa diet significantly (p<0.05) downregulated the expression of lipid genes (FAS [0.48%], ACC [0.88%]) and 3-hydroxy-3-methylglutaryl coenzyme A reductase (HMGR) (0.68%) as a cholesterol synthesis gene, while also upregulated the expression of fatty acid oxidation gene (CPT-1) (1.75%). Conversely, 1% treatment significantly downregulated (p<0.05) genes FAS (0.77%) and HMGR (0.80%).

## DISCUSSION

In the current study, dietary supplementation of BSFLO-SCa decreased FCR. Our findings differ with Patterson et al [21], who revealed that the dietary inclusion until 4.5% of BSFLO did not affect laying hen performance such as BW, ADFI, HDA, EW, and FCR. Conversely, Aprianto et al [11] showed different results, reporting that dietary supplementation 2% with BSFLO-SCa decreased feed intake, BW, performance index, and increased FCR. The reduction in FCR in this study is suspected to be due to the presence of lauric acid content in BSFLO. Lauric acid, a major component of BSFLO, has demonstrated strong antibacterial properties, particularly against Gram-positive bacteria [22]. The presence of lauric acid in BSFLO has been associated with antimicrobial effects and has been shown to positively influence poultry and gut health [23]. Anas et al [10] and Chan et al [24] reported that the inclusion of BSFLO in feed can enhance the gut health of laying hens and broilers. This is evidenced by improved intestinal morphology parameters, specifically an increased villi height-to-crypt depth ratio and upregulated expression of tight junction genes. Anas et al [10] added that this effect impacts laying performance, particularly resulting in a reduction in FCR. The FCR in laying quail performance can be reduced by using BSFLO, due to the lauric acid content as a MCFA, which enhances digestion and nutrient absorption without negatively impacting laying performance.

Research on the effect of BSFLO on physical quality of egg is still limited, while studies involving dietary BSFL meal are more frequently conducted. Dietary BSFL meal has been shown to influence the physical quality of quail eggs, resulting in increases in egg shape index, shell weight, shell percentage, and yolk color [25]. In current study, dietary supplementation with BSFLO-SCa increased EW, shape index, while decreasing the HU. Some of the benefits of the MCFA content in BSFLO, which have been previously explained, contribute to increasing EW and shape index. MCFA can optimize energy use, exhibit antimicrobial properties, and increase blood protein [11], thereby optimizing the egg formation process. The decrease in HU observed in this study occurred due to an increase in EW and a decrease in albumen height in the 2% treatment of dietary BSFLO-SCa, which had the lowest value, although not statistically significant. The formula for calculating the HU takes into account the albumen height and EW, providing a standardized measure that correlates with the freshness and quality of the egg [26]. Research has shown a significant positive correlation between the HU and albumen height, indicating that higher albumen height is associated with a higher HU. Conversely, there is a negative correlation between the HU and EW, suggesting that heavier eggs may have lower HU values [27]. Patterson et al [21] and Kim et al [28] reported that the BSFLO significantly influenced the increase in yolk color values of laying hens. This effect is thought to be due to differences in the BSFL used in oil production. The composition of BSFL can be influenced by the diet provided during rearing, particularly those containing carotenoid pigments (fruit and vegetable waste) [29]. Additionally, variations in feed composition may also contribute to differences in yolk color outcomes.

The data revealed that cholesterol and LDL-cholesterol content were significantly decreased (p<0.05) with increased supplementation of BSFLO-SCa. These results differ from those reported by Aprianto et al [11], where supplementation of 3% BSFLO-SCa only affected the increase in HDL-cholesterol, and supplementation of up to 2% did not affect broilers’ blood lipid profiles. There are three mechanisms that may contribute to this reduction. The first mechanism involves MCFA acting as a ligand that can inhibit the transcription of sterol regulatory element-binding protein 1 (SREBP-1), which regulates the transcription of the HMGR gene, thereby reducing cholesterol synthesis [30]. The second mechanism is that MCFA increases bile acid synthesis and excretion by upregulating peroxisome proliferator-activated receptors (PPAR)γ1, LXRγ, ABCA1, and ABCG8 expression in the liver, leading to decreased cholesterol concentration [31]. The third mechanism is that MCFA enhances HDL-cholesterol levels by increasing Apolipoprotein A1 secretion, which results in lower LDL-cholesterol and total cholesterol [7]. However, this study is limited to demonstrating the first mechanism, as evidenced by the decreased expression of the HMGR gene (Figure 1). On the other hand, the high SFA content in CPO given to the control treatment will cause an increase in the cholesterol content. The study conducted by Mason et al [32] presents evidence indicating that SFA in the form of C12-C16 fatty acids can elevate concentrations of both LDL-cholesterol and HDL-cholesterol, resulting in higher levels of blood cholesterol.

The present study showed that BSFLO-SCa supplementation increased the levels of protein in both yolk and albumen, while reducing the levels of yolk cholesterol, and albumen. These findings contrast with those reported by Heuel et al [33], who found that dietary supplementation of BSFL meal and oil did not affect the chemical quality of the eggs in laying hens.. The primary source of egg yolk proteins is vitellogenin, which is absorbed by developing oocytes via receptor-mediated endocytosis after being produced in the liver. Vitellogenin undergoes enzymatic processing to yield yolk proteins, which are subsequently retained in the ooplasm [34]. Egg yolk protein precursors are released into the bloodstream and transported to the ovarian follicles, where they are modified by internal protein enzymes [35]. The oviductal magnum in laying hens is the organ responsible for producing and releasing egg-white proteins, which mostly consist of ovalbumin, mucoproteins, and globulins [36]. The increased protein content in both egg yolk and albumen observed in this study can be attributed to the MCFA content in BSFLO, which can influence protein synthesis. MCFA have a more ketogenic effect compared to long chain fatty acids. They can enhance calorie expenditure and serve as a source of energy, thus reducing to utilize protein as an energy source and leading to an increase in protein concentration [36]. This study also showed a decrease in albumen ash content with 2% BSFLO-SCa supplementation. However, the reason for the decrease in albumen ash content is still not understood, necessitating further research to determine its effects and mechanisms. Cholesterol in egg yolk is primarily synthesized in the liver, secreted as very low density lipoprotein in the blood, and then deposited in the yolk through receptor-mediated endocytosis [36]. This process involves the liver synthesizing cholesterol, which is then transported to the yolk through the bloodstream. The decrease in cholesterol in egg yolks aligns with the findings of cholesterol reduction in the blood and HMGR gene expression (Figure 1). Enzymes such as HMGR play a vital role in regulating the amount of cholesterol present in egg yolks [37]. This supports the results of research conducted by Wang et al [38] after inhibiting the activity of liver HMGR with HMGR inhibitors, they observed alterations in the expression of genes related to cholesterol synthesis, bile acid synthesis, and cholesterol transport in the liver, ultimately reduces cholesterol synthesis and transports cholesterol to the egg yolk.

The findings of the current study are supplementation BSFLO-SCa downregulated lipid (FAS, ACC) and cholesterol synthesis gene expression (HMGR), while upregulating the fatty acid oxidation gene expression (CPT-1). Similarly, Aprianto et al [11] reported that supplementation with BSFLO-SCa downregulated the expression of ACC and FAS while CPT-1 was upregulated. The liver in poultry plays a crucial role in the metabolism of lipids and cholesterol, where enzymes control these processes at every stage. MCFA have been shown to downregulate the expression of key lipid genes such as FAS and ACC. MCFA have also been shown to reduce the expression and activity of SREBP-1. MCFA is involved in the upregulation of Insig-1, which can decrease SREBP-1c expression, thereby inhibiting lipid synthesis [39]. Moreover, MCFA may interfere with SREBP-1 proteolytic processing, resulting in reduced production of mature SREBP-1 and decreased lipogenic gene transcription, leading to reduced SREBP-1 protein synthesis and activity. SREBP-1 has been shown to activate the expression of various SREBP-target genes, including HMGR and FAS [40]. Additionally, MCFA act as natural ligands for PPARγ, leading to an upregulation in mRNA expression of genes implicated in fat oxidation, such as CPT-1. Moreover, MCFA upregulate adipose triglyceride lipase and hormone-sensitive lipase, which are rate-limiting enzymes involved in mobilizing fatty acids and hydrolyzing triglycerides, potentially leading to an increase in the availability of fatty acids for oxidation, including the activation of CPT-1 [41]. MCFA, as natural ligands, will influence the transcription factors of several genes by binding to specific DNA sequences called enhancers or promoters to affect the conversion of genetic information into proteins [42].

## CONCLUSION

The study found that supplementation with BSFLO-SCa reduced the FCR without negatively impacting laying performance. This supplementation also decreased cholesterol and LDL cholesterol levels in blood serum, which contributed to reduced cholesterol levels in egg yolk. Additionally, feed containing BSFLO-SCa increased protein content in the yolk and albumen. The supplementation lowered the expression of genes related to lipid and cholesterol synthesis (FAS, ACC, HMGR) while increasing the expression of genes associated with fatty acid oxidation (CPT-1) in the liver. However, further studies are needed to evaluate the effects of BSFLO-SCa on ash content and other parameters, and to compare results without saponification treatment. BSFLO-SCa could serve as an alternative oil source in poultry feed, especially for quail. Currently, BSFLO is more expensive, making it unsuitable for commercial use. Integrating farming with BSF cultivation can be implemented to support the sustainable potential of BSFLO.

## Figures and Tables

**Table 1:** Fatty acid profiles and gross energy value of black soldier fly larvae oil calcium salt (BSFLO-SCa) and crude palm oil calcium salt (CPO-SCa)

Items	BSFLO-SCa	CPO-SCa
Fatty acid profiles (%)		
Decanoic acid (C10:0)	1.34	0.79
Lauric acid (C12:0)	36.96	0.30
Myristic acid (C14:0)	7.90	1.20
Pentadecanoic acid (C15:0)	0.20	0.19
Palmitic acid (C16:0)	15.46	55.20
Heptadecanoid acid (C17:0)	0.34	0.17
Stearic acid (C18:0)	ND	4.50
Heneicosanoic acid (C21:0)	0.35	ND
Lignoceric acid (C24:0)	0.43	0.16
Palmitoleic acid (C16:1)	2.27	3.35
Oleic acid (C18:1)	0.44	15.20
Linoleic acid (C18:3)	34.36	19.82
Gamma-lonoleic acid (C18:3)	ND	0.45
Eicosanoic acid (C20:1)	1.04	ND
Alpha-linolenic acid (C18:3)	0.23	0.91
Eicosatrienoic acid (C20:0)	0.18	0.20
Eicosatetranoic acid (C18:3)	ND	ND
Nervoic acid (C24:1)	ND	0.15
SFA	62.98	62.51
MUFA	3.71	18.70
PUFA	34.77	21.38
Gross energy (Cal/g)	6,942.69	7,264.40

ND, not detected; SFA, saturated fatty acids; MUFA, monounsaturated fatty acids; PUFA, polyunsaturated fatty acids.

**Table 2:** Compositions and nutrient content of experimental diets

Feed Ingredients	BSFLO-SCa		

	T0^1)^	T1	T2
Corn	48.00	48.77	49.47
Rice bran	15.37	14.60	13.90
SBM^2)^	25.10	25.10	25.10
Meat bone meal	2.00	2.00	2.00
CPO-SCa	2.00	1.00	0.00
BSFLO-SCa	0.00	1.00	2.00
Limestone	5.80	5.80	5.80
Dicalcium phosphate	0.50	0.50	0.50
NaCl	0.40	0.40	0.40
Vitamin Mix^3)^	0.04	0.04	0.04
Mineral Mix^4)^	0.10	0.10	0.10
DL-Methionine^5)^	0.20	0.20	0.20
L-Lysine HCl^5)^	0.10	0.10	0.10
L-Threonine^5)^	0.05	0.05	0.05
L-Isoleusine^5)^	0,05	0.05	0.05
Choline chloride	0.09	0.09	0.09
Toxin binder	0.20	0.20	0.20
Total	100.00	100.00	100.00
Nutrient Content			
Crude protein (%)	20.59	20.56	20.52
Ether extract (%)	5.21	4.78	4.36
Crude fiber (%)	4.13	4.05	3.98
ME (kcal/kg)	2,923	2,923	2,923
Ca (%)	2.51	2.52	2.54
Available P (%)	0.35	0.35	0.35
Methionine (%)	0.49	0.49	0.49
Lysine (%)	1.08	1.08	1.07
Threonine (%)	0.78	0.78	0.78

1) T0, basal diet; T1, basal diet+1% BSFLO-SCa; T2, basal diet+2% BSFLO-SCa.

2) SBM contains 49,33% crude protein.

3) Supplied per kg if diet: Vitamin A, 50,000,000 IU, Vitamin D3, 10,000,0000 IU; Vitamin E, 80,000 mg; Vitamin K3, 10,000 mg; Vitamin B1, 10,000 mg; Vitamin B2, 30,000 mg, Vitamin B3, 225,000 mg; Vitamin B5, 62,000 mg, Vitamin B6, 10,000 mg; Vitamin B9, 5,000 mg; Vitamin B12, 100 mg; Vitamin H, 100 mg; Vitamin C, 20,000 mg.

4) Supplied per kg of diet: Mn, 50,000 mg; Fe, 30,000 mg; Cu, 7,500 mg; Zn, 40,000 mg; I, 755 mg; Se, 150 mg.

5) of synthetic amino acid: DL-Methionine, 98%; L-Lysine HCl, 74,42%; L-Threonine, 80%; L-Isoleusine, 90%.

BSFLO-SCa, black soldier fly larvae oil calcium salt; SBM, soybean meal; CPO-SCa, crude palm oil calcium salt; ME, metabolizable energy.

**Table 3:** Fatty acid compositions of experimental diets

Fatty acids	Proportion (% amount of fatty acid)		

	BSFLO-SCa		

	T0^1)^	T1	T2
Lauric acid (C12:0)	0.50	11.92	13.93
Myristic acid (C14:0)	0.97	3.29	3.48
Palmitic acid (C16:0)	39.61	38.66	22.93
Heptadecanoid acid (C17:0)	0.11	0.19	0.15
Stearic acid (C18:0)	4.47	5.27	3.83
Docosanoic acid (C22:0)	0.18	1.38	1.36
Lignoceric acid (C:24)	0.37	0.96	0.10
Palmitoleic acid (C16:^1)^	0.15	0.52	0.83
Oleic acid (C18:^1)^	35.80	28.81	27.85
Linoleic acid (C18:2)	15.94	3.60	22.33
Gamma-linolenic acid (C18:3)	0.47	0.70	0.52
Eicosanoic acid (C20:^1)^	0.73	0.83	0.98
Alpha-linolenic acid (C18:3)	0.20	0.27	0.24
Eicosadienoic acid (C20:2)	0.10	0.91	0.14
Eicosapentaenoate acid (C20:5)	0.17	0.37	0.29
Nervoic acid (C24:^1)^	0.23	0.52	0.49
SFA	46.21	46.04	45.88
MUFA	38.91	30.68	30.15
PUFA	16.88	5.85	23.52

1) T0, basal diet; T1, basal diet+1% BSFLO-SCa; T2, basal diet+2% BSFLO-SCa.

BSFLO-SCa, black soldier fly larvae oil calcium salt; SFA, saturated fatty acids; MUFA, monounsaturated fatty acids; PUFA, polyunsaturated fatty acids.

**Table 4:** The primers sequence for analysis of fat metabolism gene expression

Gen	Primer sequence (5′ – 3′)	Orientation	Base pairs	Amplification temperature (°C)	GenBank no.
*β-actin*	GTGTGATGGTTGGTATGGGC	Forward	225	59	L08165.1
	CTCTGTTGGCTTTGGGGTTC	Reverse			
*CPT-1*	GAAGACGGACACTGCAAAGG	Forward	213	59	AY675193.1
	GGGCAAGTTGAATGAAGGCA	Reverse			
*ACC*	GCTGGGTTGAGCGACTAATG	Forward	250	59	205505.1
	GGGAAACTGGCAAAGGACTG	Reverse			
*FAS*	TGGTTGACTGCCACCAATTG	Forward	173	59	J04485.1
	ACCCCACTTTCCATCACGAT	Reverse			
*HMGR*	TCCCTGAACCCTCATCTTTG	Forward	223	60	NM_204485
	TCTGCAAGAATACGGCTCCT	Reverse			

CPT-1, carnitine palmitoyltransferase; ACC, acetyl-Coacarboxylase; FAS, fatty acid synthase; HMGR, 3-hydroxy-3-methyl glutaryl coenzyme A reductase.

**Table 5:** Effect supplementation of BSFLO-SCa on the laying performance (n= 8 per treatment)

Parameters	BSFLO-SCa			SEM	p-value

	T0^1)^	T1	T2		
HDA (%)	79.16±8.41	84.68±2.66	78.49±9.68	1.584	0.224
EM (g)	9.13±1.02	9.8±0.49	9.18±0.97	0.179	0.249
EW (g)	11.55±0.42	11.57±0.32	11.73±0.46	0.081	0.635
ADFI (g)	26.27±0.74	26.75±0.86	27.15±1.40	0.211	0.306
FCR	2.92±0.17^a^	2.73±0.10^b^	2.77±0.13^b^	0.032	0.022

1) T0, basal diet; T1, basal diet+1% BSFLO-SCa; T2, basal diet+2% BSFLO-SCa.

a,b Means within a row with different superscripts are different (p<0.05).

BSFLO-SCa, black soldier fly larvae oil calcium salt; SEM, standard error of the mean; HDA, hen day average; EM, egg mass; EW, egg weight; ADFI, average daily feed intake; FCR, feed conversion ratio.

**Table 6:** Effect supplementation of BSFLO-SCa on the physical egg quality (n= 8 per treatment)

Parameters	BSFL-SCa			SEM	p-value

	T0^1)^	T1	T2		
Egg weight (g)	11.52±0.27^b^	11.59±0.48^b^	11.98±0.26^a^	0.081	0.033
Egg shape index (%)	77.47±1.02^b^	77.52±0.96^b^	78.61±0.65^a^	0.205	0.029
Yolk color	4.58±0.32	4.64±0.14	4.42±0.09	0.045	0.112
Yolk height (mm)	11.20±0.30	11.09±0.43	10.79±0.24	0.075	0.060
Yolk width (mm)	23.17±0.32	23.04±0.40	23.38±0.62	0.096	0.340
Albumen height (mm)	3.98±0.31	3.99±0.34	3.66±0.34	0.071	0.100
Haugh unit	86.19±1.72^a^	86.28±1.87^a^	83.92±1.94^b^	0.426	0.029
Albumen width (mm)	38.81±1.43	39.40±1.66	39.75±1.80	0.329	0.521
Eggshell weight (g)	1.30±0.05	1.31±0.05	1.28±0.09	0.013	0.689
Eggshell thickness (mm)	0.35±0.01	0.33±0.02	0.34±0.01	0.003	0.094

1) T0, basal diet; T1, basal diet+1% BSFLO-SCa; T2, basal diet+2% BSFLO-SCa.

a,b Means within a row with different superscripts are different (p<0.05).

BSFLO-SCa, black soldier fly larvae oil calcium salt; SEM, standard error of the mean.

**Table 7:** Blood lipid profile of Japanese quail fed with black soldier fly larvae oil calcium salt (BSFLO-SCa) (n= 6 treatment)

Parameter (mg/dL)	BSFL-SCa			SEM	p-value

	T0^1)^	T1	T2		
Cholesterol	172.82±9.25^a^	145.30±14.53^b^	150.08±21.99^b^	4.607	0.022
Triglyceride	1,144.85±252.37	1,059.64±145.12	1,014.45±102.870	41.589	0.456
HDL-C	16.58±3.27	15.14±5.95	17.5±2.85	0.971	0.635
LDL-C	100.55±17.57^a^	65.83±13.50^b^	55.35±16.16^b^	5.852	0.000

1) T0, basal diet; T1, basal diet+1% BSFLO-SCa; T2, basal diet+2% BSFLO-SCa.

a,b Means within a row with different superscripts are different (p<0.05).

BSFLO-SCa, black soldier fly larvae oil calcium salt; SEM, standard error of the mean; HDL-C, high density lipoprotein cholesterol; LDL, low density lipoprotein cholesterol.

**Table 8:** Effect supplementation of BSFLO-SCa on the chemical quality of egg (n= 6 per treatment)

Parameters	BSFLO-SCa			SEM	p-value

	T0^1)^	T1	T2		
Yolk					
Dry matter (%)	51.26±2.31	52.13±1.37	52.19±2.09	0.448	0.660
Ash (%)	1.82±0.29	1.87±0.29	2.07±0.27	0.068	0.297
Protein (%)	16.62±0.55^b^	16.14±0.65^b^	18.84±1.77^a^	0.379	0.002
Fat (%)	27.49±2.11	27.64±2.15	27.15±2.28	0.485	0.923
Cholesterol (mg/100 g)	1,594.30±163.65^a^	1,494.19±181.40^a^	1,247.46±96.39^b^	51.223	0.010
Albumen (%)					
Dry matter	12.98±0.47	12.78±0.32	12.46±0.93	0.149	0.385
Ash	0.76±0.14^a^	0.70±0.14^a^	0.54±0.15^b^	0.036	0.017
Protein	11.37±0.62^b^	11.23±0.52^b^	12.66±0.52^a^	0.199	0.001

1) T0, basal diet; T1, basal diet+1% BSFLO-SCa; T2, basal diet+2% BSFLO-SCa.

a,b Means within a row with different superscripts are different (p<0.05).

BSFLO-SCa, black soldier fly larvae oil calcium salt; SEM, standard error of the mean.
